# Supplementary material for: Hybrid Models and Biological Model Reduction with PyDSTool
Source: PLoS Comput Biol. 2012 Aug 9;8(8):e1002628. doi: 10.1371/journal.pcbi.1002628 (PMC3415397; doi:10.1371/journal.pcbi.1002628)
Supplement: Text S4 — Complete source code for the PyDSTool package (version 0.88.120504). Includes API documentation and help files linking to web pages. This file is identical to the current public release on Sourceforge.net. (ZIP) [file pcbi.1002628.s004.zip › PyDSTool/html/PyDSTool.Toolbox.DSSRT_tools-pysrc.html]

xml version="1.0" encoding="ascii"?


PyDSTool.Toolbox.DSSRT\_tools


| Home | Trees | Indices | Help | PyDSTool |
| --- | --- | --- | --- | --- |

Package PyDSTool ::
Package Toolbox ::
Module DSSRT\_tools

[hide private]

[frames] | no frames]

# Source Code for Module PyDSTool.Toolbox.DSSRT\_tools

```
  1  # DSSRT interface tools (incomplete)
 
  2  """DSSRT interface tools.
 
  3  (strongly biased towards neural models at this time).
 
  4  
 
  5  These utilities do not fully construct the graphical
 
  6  specification for DSSRT, which still has to be done by hand.
 
  7  Partial templates for the graphical specs are created.
 
  8  
 
  9     Robert Clewley, October 2005.
 
 10  
 
 11  """ 
 12  
 
 13  from PyDSTool import * 
 14  from PyDSTool.parseUtils import symbolMapClass 
 15  from FR import * 
 16  import os 
 17  from copy import copy 
 18  from random import uniform, gauss 
 19  
 
 20  __all__ = ['DSSRT_info', 'plotNetworkGraph'] 
 21  
 
 22  # ---------------------------------------------------------------------------
 
 23  
 
 24  CFGorder = ['VARSEXT', 'VARSINT', 'INPUTS', 'DEQNS', 'CFAC', 'BOUNDS',
 
 25              'UNITBOUNDS', 'GAM1TERM', 'GAM2TERM', 'DEPAR'] 
 26  
 
 27  nodes_order = ['NODE', 'NODELABEL', 'NODEADDSTATES', 'NODESTATEMAP_ACTS',
 
 28                 'NODESTATEMAP_POTS'] 
 29  
 
 30  
 


31 -class DSSRT_info(object):


32 -    def __init__(self, name='', infty_val=1e10):


33          """The optional 'infty_val' argument sets the substitute default
 
 34          finite value used to represent 'infinity', for variables that are
 
 35          not given explicit bounds.""" 
 36  
 
 37          self.name = name 
 38          self.varsext = [] 
 39          self.varsint = [] 
 40          self.varsall = [] 
 41          self.deqns = {} 
 42          self.depars = {} 
 43          self.inputs = {} 
 44          self.bounds = {} 
 45          self.unitbounds = [] 
 46          self.gam1terms = {} 
 47          self.gam2terms = {} 
 48          self.cfac = {} 
 49          # graphics templates
 
 50          self.nodes = [] 
 51          self.links = [] 
 52          self.vbars = [] 
 53          # for internal use
 
 54          self._CFG = {} 
 55          self._infty_val=infty_val

 56  
 
 57  
 


58 -    def setUnitBounds(self, coords):


59          self.unitbounds = coords

 60  
 
 61  
 


62 -    def prepInputs(self, dependencies, nameMap=None):


63          if nameMap is None: 
 64              nameMap = symbolMapClass({}) 
 65          inputs = {}.fromkeys(self.varsall) 
 66          for (i,o) in dependencies: 
 67              mi = nameMap(i) 
 68              mo = nameMap(o) 
 69              if mi != mo: 
 70                  try: 
 71                      inputs[mi].append(mo) 
 72                  except AttributeError: 
 73                      inputs[mi] = [mo] 
 74          for k, v in inputs.iteritems(): 
 75              if v is None: 
 76                  inputs[k] = [] 
 77          self.inputs = inputs

 78  
 
 79  
 


80 -    def prepGraph(self, ics=None, vbars_int={}, vars=None):


81          """Call this multiple times to re-randomize the initial vertex
 
 82          selection.""" 
 83          if self.varsall == [] or self.inputs == {}: 
 84              raise ValueError("Prerequisite of prepGraph is varsall and inputs") 
 85          if vars is None: 
 86              vars = self.varsext 
 87          # TEMP  -- only support full vars (no hidden)
 
 88          if remain(self.varsext, vars) != []: 
 89              raise NotImplementedError("Can only make network graph with all "
 
 90                                        "external variables at this time") 
 91          print "Finding graph layout for network..." 
 92          V = {} 
 93          E = {} 
 94          # prepare vertices
 
 95          for var in vars: 
 96              V[var] = vertex(uniform(0.2, 0.8), uniform(0.2, 0.8)) 
 97  #            print "vertex %s initialized at co-ords (%.4f, %.4f)"%(var,
 
 98  #                                    V[var].pos[0], V[var].pos[1])
 
 99          # valid domain, in [0, 1]
 
100          domY = [0.08, 0.92] 
101          domX = [0.08, 0.96] 
102          # override position initial conditions for specially selected variables
 
103          if ics is not None: 
104              for var, ic in ics.items(): 
105                  if ic[0] < domX[0] or ic[0] > domX[1]: 
106                      raise ValueError("X initial condition for %s out of "%var \
 
107                                       + "bounds [%.3f, %.3f]"%(domX[0], domX[1])) 
108                  if ic[1] < domY[0] or ic[1] > domY[1]: 
109                      raise ValueError("Y initial condition for %s out of "%var \
 
110                                       + "bounds [%.3f, %.3f]"%(domY[0], domY[1])) 
111                  try: 
112                      V[var].pos = array(ic, Float) 
113                  except KeyError: 
114                      raise KeyError("Variable name %s in initial condition"%var\
 
115                                     + " dictionary not known") 
116          # prepare edges
 
117          for var in vars: 
118              ins = self.inputs[var] 
119              if len(ins) >= 1: 
120                  for invar in ins: 
121                      if invar == var: 
122                          raise ValueError("Internal error: invar == var") 
123                      try: 
124                          e = edge(V[invar], V[var]) 
125                      except KeyError: 
126                          # assume connected to an internal variable, so ignore
 
127                          continue 
128                      try: 
129                          E[var].append(e) 
130                      except KeyError: 
131                          E[var] = [e] 
132          in_d = in_degree(V, E) 
133          out_d = out_degree(V, E) 
134          for v in vars: 
135              if in_d[v] + out_d[v] == 0: 
136                  info(in_d, 'in-degrees') 
137                  print "\n" 
138                  info(out_d, 'out-degrees') 
139                  raise ValueError("Variable %s had no associated edges"%v) 
140          self._V = V 
141          self._E = E 
142          if ics is None: 
143              fixedICs = {} 
144          else: 
145              fixedICs = dict(zip(ics.keys(),[V[k] for k in ics.keys()])) 
146          # call the Fruchtermann-Reingold algorithm to determine graph
 
147          FR(V, E, domX, domY, fixed=fixedICs) 
148          self.nodes = [] 
149          self.links = [] 
150          self.vbars = [] 
151          importance = {} 
152          for var in vars: 
153              # get all importance info first
 
154              try: 
155                  importance[var] = in_d[var]/float(out_d[var]) 
156              except ZeroDivisionError: 
157                  importance[var] = 5   # arbitrary > 3 
158          # generate node info for DSSRT from FR output
 
159          for var in vars: 
160              if importance[var] > 3: 
161                  # "important" variables get larger labels, nodes
 
162                  r2 = 0.04 
163                  txtsize = 0.02 
164              else: 
165                  r2 = 0.03 
166                  txtsize = 0.015 
167              nx = V[var].pos[0] 
168              ny = V[var].pos[1] 
169              if txtsize == 0.02: 
170                  lx = nx-0.25*r2*len(var) 
171                  ly = ny-0.4*txtsize 
172              else: 
173                  lx = nx-0.27*r2*len(var) 
174                  ly = ny-0.35*txtsize 
175              self.nodes.append({'NODE': "%s %.4f %.4f %.3f"%(var, nx, ny, r2),
 
176                            'NODELABEL': "%s %s %.4f %.4f %.3f"%(var, var,
 
177                                                  lx, ly, txtsize),
 
178                            'NODEADDSTATES': "%s 0"%var,
 
179                            'NODESTATEMAP_ACTS': var, 'NODESTATEMAP_POTS': var}) 
180              x2 = V[var].pos[0] 
181              y2 = V[var].pos[1] 
182              for invar in self.inputs[var]: 
183                  if invar in self.varsint: 
184                      continue 
185                  if importance[invar] > 3: 
186                      r1 = 0.04 
187                  else: 
188                      r1 = 0.03 
189                  x1 = V[invar].pos[0] 
190                  y1 = V[invar].pos[1] 
191                  D = [x1-x2, y1-y2] 
192                  sgnx = sign(D[0]) 
193                  sgny = sign(D[1]) 
194                  try: 
195                      theta2 = atan(abs(D[1])/abs(D[0])) 
196                  except ZeroDivisionError: 
197                      theta2 = pi_/2. 
198                  link_x1 = x1-sgnx*r1*cos(theta2) 
199                  link_y1 = y1-sgny*r1*sin(theta2) 
200                  link_x2 = x2+sgnx*r2*cos(theta2) 
201                  link_y2 = y2+sgny*r2*sin(theta2) 
202                  link_str = '%s %s %.4f %.4f %.4f %.4f'%(invar, var,
 
203                             link_x1, link_y1, link_x2, link_y2) 
204                  if len(self.inputs[var]) <= 1: 
205                      # link not shown for <=1 input
 
206                      # link declaration needs 1 in final arg to suppress display
 
207                      link_str += ' 1' 
208                  self.links.append(link_str) 
209              try: 
210                  bd0 = self.bounds[var][0] 
211                  bd1 = self.bounds[var][1] 
212              except KeyError: 
213                  # expect unit bounds instead
 
214                  if var in self.unitbounds: 
215                      bd0 = 0 
216                      bd1 = 1 
217                  else: 
218                      print "Warning: variable %s has not been given bounds"%var 
219                      print " (assuming +/- 'infty_val' attribute)" 
220                      bd0 = -self._infty_val 
221                      bd1 = self._infty_val 
222              magopt = int(bd0 == 0 and bd1 == 1) 
223              self.vbars.append('%s %.4f %.4f %.4f %.3f %.3f %d'%(var,
 
224                                   x2-r2*1.2, y2+0.025, r2, bd0, bd1, magopt)) 
225          for var in remain(self.varsall, vars): 
226              try: 
227                  bd0 = self.bounds[var][0] 
228                  bd1 = self.bounds[var][1] 
229              except KeyError: 
230                  # expect unit bounds instead
 
231                  if var in self.unitbounds: 
232                      bd0 = 0 
233                      bd1 = 1 
234                  else: 
235                      print "Warning: variable %s has not been given bounds"%var 
236                      print " (assuming +/- 'infty_val' attribute)" 
237                      bd0 = -self._infty_val 
238                      bd1 = self._infty_val 
239              # these variables still need vbars
 
240              magopt = int(bd0 == 0 and bd1 == 1) 
241              if var in vbars_int: 
242                  if type(vbars_int[var])==str: 
243                      # use vertex of associated external variable
 
244                      assocV = V[vbars_int[var]] 
245                      x1 = assocV.pos[0]-0.02 
246                      y1 = assocV.pos[1]+0.025 
247                      h = 0.03 
248                  else: 
249                      # assume (x,y,h) given explicitly as a triple
 
250                      x1, y1, h = vbars_int[var] 
251                  self.vbars.append('%s %.4f %.4f %.4f %.3f %.3f %d'%(var, x1,
 
252                                               y1, h, bd0, bd1, magopt)) 
253              else: 
254                  self.vbars.append('%s <x1> <y1> <h> %.3f %.3f %d'%(var, bd0,
 
255                                                         bd1, magopt))

256  
 
257  
 


258 -    def makeDefaultGraphics(self):


259          for v in remain(self.varsext, self.nodes): 
260              self.nodes.append({'NODE': "%s <xpos> <ypos> <r>"%v,
 
261                            'NODELABEL': "%s %s <xpos> <ypos> <size>"%(v,v),
 
262                            'NODEADDSTATES': "%s 0"%v,
 
263                            'NODESTATEMAP_ACTS': v, 'NODESTATEMAP_POTS': v}) 
264          for v in remain(self.varsext, self.links): 
265              for i in self.inputs[v]: 
266                  self.links.append('%s %s <x1> <y1> <x2> <y2>'%(v,i)) 
267          for v in remain(self.varsint, self.vbars): 
268              try: 
269                  bd0 = self.bounds[v][0] 
270                  bd1 = self.bounds[v][1] 
271              except KeyError: 
272                  # expect unit bounds instead
 
273                  if v in self.unitbounds: 
274                      bd0 = 0 
275                      bd1 = 1 
276                  else: 
277                      raise NameError("Variable "+var+" has no declared bound") 
278              self.vbars.append('%s <xpos> <ypos> <height> %.3f %.3f <mag>'%(v,
 
279                                 bd0,bd1))

280  
 
281  
 


282 -    def makeDSSRTcfg(self, model, gen_name, cfg_filename):


283          """Make DSSRT configuration file from a PyDSTool Generator object that
 
284          is embedded in a Model object.""" 
285  
 
286          try: 
287              gen = model.registry[gen_name] 
288          except KeyError: 
289              raise ValueError("Generator '%s' not found in Model '%s'"%(gen_name, model.name)) 
290          except AttributeError: 
291              raise TypeError("Invalid Model object passed to makeDSSRTcfg()") 
292          try: 
293              mspec = model._mspecdict[gen_name] 
294          except (AttributeError, KeyError): 
295              raise ValueError("Model must contain ModelSpec information to proceed.") 
296          self.name = gen_name 
297          self.prepVarNames(model._FScompatibleNames(model.obsvars),
 
298                            model._FScompatibleNames(model.intvars)) 
299          assert remain(model._FScompatibleNames(model.allvars), self.varsext+self.varsint) == [] 
300          assert remain(self.varsext+self.varsint, model._FScompatibleNames(model.allvars)) == [] 
301          self.prepInputs(gen.funcspec.dependencies, model._FScompatibleNames) 
302          self.deqns = remain(self.varsall, model._FScompatibleNames(gen.funcspec.auxvars)) 
303          # Bounds
 
304          fsDict = mspec.funcSpecDict['vars'] 
305          for v in self.varsall: 
306              domain_interval = copy(fsDict[model._FScompatibleNamesInv(v)].domain[2]) 
307              if not isfinite(domain_interval[0]): 
308                  domain_interval[0] = -self._infty_val 
309              if not isfinite(domain_interval[1]): 
310                  domain_interval[1] = self._infty_val 
311              self.bounds[v] = domain_interval 
312          # Capacitance-like parameters
 
313          cvars = model.searchForNames('soma.C')[gen_name] 
314          for fullname in cvars: 
315              parts = fullname.split('.') 
316              assert len(parts)==2, "Only know how to deal with two-tier hierarchical variable names" 
317              try: 
318                  voltname = model._FScompatibleNames(model.searchForVars(parts[0]+'.'+'V')[0]) 
319              except: 
320                  print "Problem finding membrane voltage name in model spec" 
321                  raise 
322              self.cfac[voltname] = model._FScompatibleNames(fullname) 
323          # need to take out function-specific parameters from depars --
 
324          # assume that any parameters appearing in function definitions *only*
 
325          # appear there
 
326          subsFnDef, not_depars = self.prepAuxFns(mspec.flatSpec['auxfns'],
 
327                                                  mspec.flatSpec['pars']) 
328          # prepare DEpars
 
329          alldepars = model._FScompatibleNames(model.query('parameters'))   # full list of pars 
330          depar_names = remain(alldepars, not_depars) 
331          self.depars = dict(zip(depar_names, [mspec.flatSpec['pars'][p] for p in depar_names])) 
332          # perform the textual subs for non-tau or inf function calls with > 1 argument
 
333          raise NotImplementedError("This function is incomplete!") 
334          # do something with subsFnDef
 
335          # work out gam1terms and gam2terms
 
336          # validate CFG
 
337          self.prepCFG() 
338          print "Finished preparing CFG information. Call outputCFG(filename) to output .cfg file"

339  
 
340  
 


341 -    def prepCFG(self):


342          """Prepare .cfg file (at least a pre-cursor for later editing by hand)""" 
343          # Set remaining bounds to default limits
 
344          bd_overlap = intersect(self.bounds.keys(), self.unitbounds) 
345          if bd_overlap != []: 
346              print bd_overlap 
347              raise ValueError("Clash between variables with explicitly declared"
 
348                               "bounds and those with unit bounds") 
349          for v in remain(self.varsall, self.bounds.keys()+self.unitbounds): 
350              self.bounds[v] = [-self._infty_val, self._infty_val] 
351          self._CFG['VARSEXT'] = [" ".join(self.varsext)] 
352          self._CFG['VARSINT'] = [" ".join(self.varsint)] 
353          self._CFG['INPUTS'] = [ename + " " + " ".join(inlist) for ename, inlist in self.inputs.items()] 
354          self._CFG['BOUNDS'] = [ename + " %f %f"%(bd[0],bd[1]) for ename, bd in self.bounds.items()] 
355          self._CFG['UNITBOUNDS'] = [" ".join(self.unitbounds)] 
356          self._CFG['DEQNS'] = [" ".join(self.deqns)] 
357          self._CFG['CFAC'] = [vname + " " + cname for (vname, cname) in self.cfac.items()] 
358          self._CFG['GAM1TERM'] = [] 
359          for vname, termlists in self.gam1terms.iteritems(): 
360              self._CFG['GAM1TERM'].extend([vname + " " + " ".join(termlist) for termlist in termlists]) 
361          self._CFG['GAM2TERM'] = [] 
362          for vname, termlists in self.gam2terms.iteritems(): 
363              self._CFG['GAM2TERM'].extend([vname + " " + " ".join(termlist) for termlist in termlists]) 
364          deparnames = self.depars.keys() 
365          deparnames.sort() 
366          self._CFG['DEPAR'] = [] 
367          for parname in deparnames: 
368              pval = self.depars[parname] 
369              self._CFG['DEPAR'].append(parname + " " + str(pval)) 
370          # Graphics configuration templates
 
371          if self.nodes == [] or self.links == [] or self.vbars == []: 
372              self.makeDefaultGraphics() 
373          self._CFG['graphics'] = (self.nodes, self.links, self.vbars)

374  
 
375  
 


376 -    def outputCFG(self, cfg_filename):


377          if self._CFG == {}: 
378              self.prepCFG() 
379              if self._CFG == {}: 
380                  raise RuntimeError("CFG dictionary was empty!") 
381          cfg_file = open(cfg_filename+".cfg", 'w') 
382          cfg_file.write("# Auto-generated CFG file for PyDSTool model %s\n"%self.name) 
383          for k in CFGorder: 
384              v = self._CFG[k] 
385              cfg_file.write("\n### %s configuration\n"%k.title()) 
386              if v == []: 
387                  cfg_file.write("# (EMPTY)\n") 
388              for ventry in v: 
389                  cfg_file.write("%s %s\n"%(k,ventry)) 
390          try: 
391              ginfo = self._CFG['graphics'] 
392              cfg_file.write("\n### Nodes") 
393              for node in ginfo[0]: 
394                  cfg_file.write("\n") 
395                  for k in nodes_order: 
396                      cfg_file.write("%s %s\n"%(k,node[k])) 
397              cfg_file.write("\n### Links\n") 
398              for link in ginfo[1]: 
399                  cfg_file.write("LINK %s\n"%link) 
400              cfg_file.write("\n### Vbars\n") 
401              for vbar in ginfo[2]: 
402                  cfg_file.write("VBAR %s\n"%vbar) 
403          except KeyError: 
404              pass 
405          cfg_file.close()

406  
 
407  
 


408 -    def prepVarNames(self, extvars, intvars):


409          self.varsext = copy(extvars) 
410          self.varsext.sort() 
411          self.varsint = copy(intvars) 
412          self.varsint.sort() 
413          self.varsall = self.varsext+self.varsint

414  
 
415  
 


416 -    def prepDEpars(self):


417          updates = {} 
418          for par, parval in self.depars.iteritems(): 
419              try: 
420                  if par[-3:]=="tau": 
421                      updates[par] = (par+"_recip", 1/parval) 
422              except IndexError: 
423                  pass 
424          for par, (newpar, newval) in updates.iteritems(): 
425              del self.depars[par] 
426              self.depars[newpar] = newval

427  
 
428  
 


429 -    def prepAuxFns(self, auxfndict, pardict, makeMfiles=True):


430          """Prepare auxiliary functions, and create MATLAB m-files for
 
431          those corresponding to 'tau' and 'inf' functions, unless optional
 
432          makeMfiles==False.""" 
433          # DSSRT cannot accept m-file fns that involve DEpars not explicitly
 
434          # passed as an argument, hence not_depars is returned to caller
 
435          # indicating instances of this occuring
 
436          subsFnDef=[] 
437          not_depars=[] 
438          mfiles = {} 
439          allpars = pardict.keys() 
440          for fname, (fsig, fdef) in auxfndict.iteritems(): 
441              if len(fsig) == 1 and fname[-3:] in ['tau', 'inf']: 
442                  fdefQS = QuantSpec("__fdefQS__", fdef) 
443                  # fpars are the parameters used in the function -- we fetch
 
444                  # their values and subs them directly into the M file function.
 
445                  fpars = intersect(fdefQS.freeSymbols, allpars) 
446                  fpar_defs = dict(zip(fpars,[pardict[p] for p in fpars])) 
447                  not_depars.extend(remain(fpars, not_depars)) 
448                  if fname[-3:] == 'tau': 
449                      fname += "_recip" 
450                      finfo = {fname: str(1/fdefQS)}  # take reciprocal 
451                  else: 
452                      finfo = {fname: str(fdefQS)} 
453                  finfo.update(fpar_defs) 
454                  mfiles[fname] = (fsig[0], finfo) 
455              else: 
456                  # will textually substitute these function call into spec later
 
457                  subsFnDef.append(fname) 
458          # make m files if nofiles==False
 
459          if makeMfiles: 
460              for fname, finfo in mfiles.iteritems(): 
461                  makeMfileFunction(fname, finfo[0], finfo[1]) 
462          return subsFnDef, not_depars

463  
 
464  
 


465 -    def dumpTrajData(self, traj, dt, filename, precise=True):


466          """If precise=True (default), this trajectory dump to file may take
 
467          several minutes:
 
468          Uses the 'precise' trajectory sample option for trajectories with
 
469          variable time-steps. Use precise=False option for
 
470          trajectories calculated at fixed time steps.""" 
471          ptset = traj.sample(dt=dt, coords=self.varsall, precise=precise) 
472          exportPointset(ptset, {filename: tuple(['t']+self.varsall)})

473  
 
474  
 
475  # ------------------------------------------------------------------------
 
476  
 


477 -def plotNetworkGraph(dssrt_obj):


478      try: 
479          V = dssrt_obj._V 
480          E = dssrt_obj._E 
481      except AttributeError: 
482          raise TypeError("Invalid DSSRT_info object for plotNetworkGraph") 
483      pylab.figure() 
484      for v in V.itervalues(): 
485          pylab.plot([v.pos[0]],[v.pos[1]],'ko') 
486      for elist in E.itervalues(): 
487          for e in elist: 
488              pylab.plot([e.u.pos[0],e.v.pos[0]],
 
489                         [e.u.pos[1],e.v.pos[1]],
 
490                         'k-') 
491      pylab.axis([0,1,0,1])

492
```

  


| Home | Trees | Indices | Help | PyDSTool |
| --- | --- | --- | --- | --- |

|  |  |
| --- | --- |
| Generated by Epydoc 3.0.1 on Fri May 4 15:24:25 2012 | http://epydoc.sourceforge.net |
